# Adaptation and Resilience: Lessons Learned From Implementing a Combination Health and Education Intervention for Adolescent Girls and Young Women in South Africa During the COVID-19 Pandemic

**DOI:** 10.3389/frhs.2022.903583

**Published:** 2022-06-03

**Authors:** Zoe Duby, Brittany Bunce, Chantal Fowler, Kim Jonas, Darshini Govindasamy, Colleen Wagner, Kgahliso Mangoale, Anthony Ambrose, Catherine Mathews

**Affiliations:** ^1^Health Systems Research Unit, South African Medical Research Council, Cape Town, South Africa; ^2^Division of Social and Behavioural Sciences in the School of Public Health and Family Medicine, University of Cape Town, Cape Town, South Africa; ^3^Institute for Global Sustainable Development (IGSD), University of Sheffield, Sheffield, United Kingdom; ^4^NACOSA (Networking HIV/AIDS Community of South Africa), Cape Town, South Africa

**Keywords:** COVID-19, South Africa, health system resilience, implementation, sexual and reproductive health (SRH), adolescent girls and young women (AGYW), combination intervention

## Abstract

The COVID-19 pandemic has been associated with reduced access to health services and worsening health outcomes for HIV and sexual and reproductive health (SRH). Through the analysis of data from an evaluation study of a combination intervention for adolescent girls and young women (AGYW) in South Africa, we sought to examine the way in which implementation and service provision were impacted by the COVID-19 pandemic and related restrictions, describing the adaptation implementers made to respond to this context. The intervention was implemented from 2019 in South African districts identified as high priority, given the high rates of HIV and teenage pregnancy amongst AGYW. The South African government introduced the first COVID-19 lockdown in March 2020. We conducted in-depth interviews with 38 intervention implementers in the period from November 2020 to March 2021. Respondents described various ways in which the COVID-19 pandemic and related restrictions had limited their ability to implement the intervention and provide services as planned. As a result, AGYW intervention beneficiary access to SRH and psychosocial services was disrupted. Implementers described several ways in which they attempted to adapt to the pandemic context, such as offering services remotely or door-to-door. Despite attempts to respond to the context and adapt services, overall COVID-19 negatively affected implementation and service provision, and heightened issues around community acceptability of the programs. Our findings can help to inform efforts to reduce health service disruption, increase health system resilience, and ensure continuous SRH service provision to AGYW in times of pandemics and other crises.

## Introduction

### Background to the Intervention

In South Africa, adolescent girls and young women (AGYW) account for over 67% of new HIV infections, acquiring HIV at twice the rate of their male counterparts (1). Additionally, estimates suggest that 30% of AGYW aged between 10 and 19 years in South Africa experience pregnancy, with over 65% of these pregnancies being unintended (2). South Africa's National Strategic Plan, 2017–2022 singled out AGYW given their “extraordinarily high incidence of HIV” (3). Since the disproportionate HIV risks faced by AGYW are due to a range of social and structural inequities that shape and constrain HIV-risk behaviors, comprehensive multilevel HIV prevention interventions comprising behavioral and structural interventions that aim to address individual, social, and structural drivers of HIV, in addition to biomedical interventions, have been identified as a critical action in efforts to reduce HIV infections (1, 4). Combination HIV prevention and care interventions, inclusive of biomedical, behavioral and structural components, are one of the key strategies for reaching the 90-90-90 targets and achieving the Sustainable Development Goal (SDG) of ending the HIV epidemic by 2030 (4, 5). In response to emerging epidemiological data reflecting the enormously disproportionate burden of HIV on AGYW, the Global Fund to Fight AIDS, TB and Malaria invested in a new program led by the South African government and supported by global health partners. The goal was to implement a program encompassing long-term measures to address structural barriers that enhance AGYW vulnerability and disproportionate risk, and the intervention's specific aims were to increase retention in school, decrease HIV incidence, decrease teenage pregnancy, decrease gender-based violence and increase economic opportunities for AGYW aged 15–24 in 12 high priority districts in South Africa using a combination of structural, behavioral and biomedical approaches.

Implementation of the intervention began in 2019, managed by three non-governmental organizations (NGOs) functioning as “Principal Recipient” (PR) organizations. These organizations sub-contracted additional NGOs to implement the intervention components, functioning as “Sub-Recipients” (SRs) of the grant. Implementers received support from the South African National AIDS Council (SANAC) through the Country Coordinating Mechanism (CCM). The intervention design conceived that with guidance from the CCM, Technical Advisors and SANAC-convened technical working groups (TWGs), PRs would engage government departments such as Department of Health (DOH), Department of Basic Education (DBE), Department of Social Development (DSD) and Department of Women, Youth and Persons with Disabilities at national, provincial and district-levels, facilitating access and collaborative relationships. PRs would work closely with district support partners, local SRs, and existing community and faith based organizations, to engage with and reach AGYW by operationalizing the program.

AGYW were introduced to the intervention through a number of entry points and referred to receive services via two main service components called the “core service”, received first, and “layered services”, additional biomedical, behavioral and structural services depending on the needs of the beneficiary, to be delivered over time. The core intervention services consisted of three main activities: demand creation, risk and vulnerability assessments and individualized follow-up service plans. Core services also included HIV, TB and gender-based violence (GBV) screening, the offer of HIV testing and male and female condoms, and HIV, TB, STI, and GBV information. Core services were intended to be delivered in schools, Technical Vocational Education and Training (TVET) colleges and community safe spaces, and provided to each AGYW every 6 months. Based on identified risks and needs, the program tailored a set of behavioral, biomedical and structural services in the form of “layered services”, to ensure each AGYW received services responsive to her specific risks and needs. Layered Services included comprehensive biomedical services from mobile or fixed clinics in/near schools and in communities, behavioral services delivered at safe spaces and other settings in communities, and structural services delivered at safe spaces and other settings in communities focused on AGYW but also on changing norms and raising awareness of GBV among men, boys, parents, and caregivers.

### COVID-19 Pandemic Context

Lessons from prior health crises have demonstrated that the impact of an epidemic on a population's SRH remain poorly understood and ill-recognized, due to the fact that these impacts are not directly caused by the epidemic's characteristic infectious disease, but rather due to SRH service provision being disrupted when health systems are under pressure (6, 7). Past humanitarian crises have led to reduced access to services for contraceptives, termination of pregnancy, antenatal care, HIV testing and treatment, GBV, and mental health care services; similarly the COVID-19 pandemic has been associated with reduced access to health services and a worsening in health outcomes for HIV and SRH, adversely affecting women and girls' access to SRH services including prevention commodities, contraceptives, HIV and STI care (8–11). Across the globe, COVID-19 lockdown restrictions, a prioritization of health funds for the pandemic, disruptions to the manufacture and supply of SRH commodities, redeployment of health workers to the frontline response, absenteeism of health workers due to fear of infection or illness, suspension of “non-essential” services, service closures and fear of contracting COVID-19 at health facilities, have resulted in reduced access to SRH and social protection services (10, 12–14). Additionally, people already on the margins of society, whose human rights are least protected, such as women and adolescents, are likely to be disproportionately affected by the lack of access to routine health care, as well as the economic and social consequences of the COVID-19 pandemic, as pre-existing inequalities and health disparities are amplified (6, 9, 11, 12). Evidence suggests that the COVID-19 pandemic has led to a sharp decline in SRH service access for adolescent girls and young women (AGYW) in low- and middle-income countries, consequentially leading to adverse impacts on AGYW's access to health and psychosocial services, as well as social protection (12). Estimates suggest that early pregnancies have increased by as much as 65% in member states of the Southern African Development Community (15).

In response to the first diagnosed case of SARS-CoV-2 (COVID-19) in South Africa in March 2020, the government declared a national state of disaster and announced an initial 21-day nationwide “lockdown” (16, 17). During the rest of 2020, and into 2021, a series of lockdowns and restrictions of varying intensity, or “levels” were implemented, in response to the pandemic “waves” and number of COVID-19 infections (16). The five-level COVID-19 alert system introduced by the South African government followed a risk-adjusted approach guided by criteria such as the level of infections and rate of transmission, the capacity of health facilities, and the extent of the implementation of public health interventions. “Alert Level 1” indicating a low virus transmission with “high health system readiness”; “Alert Level 5” indicating a high virus transmission with “low health system readiness”.^1^ Several waves of COVID-19, and the ensuing lockdowns and government imposed restrictions, have significantly disrupted existing health services and interventions, with interruptions in supply chains, diversion of resources, patient and provider fears of infection, transport challenges, closure of facilities, staff shortages, paring down of services, and stock outs of medicines and commodities (17–19). In terms of SRH services, contraceptive access was hampered, fewer people were tested for HIV and consequently fewer people were initiated on HIV treatment, and access to counseling, maternal and child health services were limited (17, 19).

The South African government's monitoring data shows that the number of women and girls receiving contraceptives was lower in 2020 than in the previous year, and emerging evidence points toward increased HIV infection rates amongst AGYW since the start of the pandemic (20, 21). Disruptions in the provision of sexual and reproductive health (SRH) services to South African AGYW caused by COVID-19 are likely to have exacerbated existing challenges and barriers to access faced by this population, resulting in increased adolescent pregnancies and HIV-infections (19). In recognition that continuous provision of health services, including SRH services, is vital in order to support and improve AGYW health, South African health service providers and implementing organizations have attempted to adapt to the pandemic context through various means, including increasing the services available remotely (telemedicine), and expanding medicine dispensary and commodity provision options (17). However, despite these modifications and adaptations, the provision of routine health services across South Africa was negatively impacted (17).

This study aimed to examine the extent to which the context of the intervention has affected implementation, with a specific focus on the COVID-19 pandemic and related restrictions. In the analysis presented in this paper we sought to examine the way in which a South African combination SRH intervention for AGYW was impacted by the COVID-19 pandemic and related restrictions, and describe the strategies implementers employed to adapt to this context. For frameworks with which to interpret our findings, we used the concepts of health system resilience, alongside the concept of adaptation and modification. Adaptation and modification have refer to a process of either deliberate or proactive alteration to an intervention, in order to improve effectiveness or appropriateness, to react to an unanticipated event or given context (22). The concept of resilience refers to the ability of health systems to effectively respond to crises and shocks, while maintaining core functions, and based on lessons learned, dynamically adapt and modify the system to respond to context (23). Health system crises brought on by pandemics such as COVID-19 provide a platform through which to learn and strengthen health systems, increasing their preparedness and ability to adapt to contextual needs, enabling them to effectively respond to crisis scenarios, whilst ensuring continuity in service provision (23–25). We use an adapted FRAME reporting method (22) to present findings on adaptations and modifications made to the intervention. By assessing adaptation and resilience strategies adopted by implementers in this context, our findings can help to provide evidence with which understand how to ensure SRH health system resilience in times of pandemics and other major events. We discuss the adaptations implementers made to respond to this context, to inform the implementation of similar interventions in the future.

## Methods

The HERStory2 study^2^ was a process evaluation of the AGYW combination intervention, conducted by an external evaluation team. The sample was drawn from 6 of the 12 sub-districts in which the intervention was being implemented, comprising two sub-districts per PR, as follows: Klipfontein, Cape Town (Western Cape), King Cetshwayo (KwaZulu Natal), Ehlanzeni (Mpumalanga), Rustenburg (North West), Nelson Mandela Bay (Eastern Cape), and Thabo Mofutsanyana/Dihlabeng (Free State). The sample included 38 implementer respondents, comprising 27 program managers and facilitators, four healthcare workers, and seven social workers.

Individual in-depth interviews (IDIs) were conducted telephonically in the period from November 2020 to March 2021, following semi-structured interview topic guides. Interviews explored contextual factors that may have shaped the delivery of the intervention, specifically focusing on the ways in which the context of the COVID-19 pandemic and related lockdown restrictions impacted implementation of the intervention. We asked respondents to describe contextual factors that affected implementation, with a specific focus on how shocks and stressors related to COVID-19 affected implementation. A multi-lingual team of female social scientists, who were independent from the intervention implementation, and had received training on the protocol, study specific methods and research tools, and human subject research ethics, conducted interviews in participants' language of choice. Interviews were audio-recorded with participants' consent, transcribed verbatim or translated into English as necessary, and reviewed for accuracy.

Analysis was conducted by a team of three analysts, using an iterative thematic approach. Verbatim or translated transcripts were analyzed first through identification of emergent key themes and topics in initial readings. The analysis process evolved iteratively through a deductive and inductive process, reflecting on the study's key objectives and topics that emerged through reviewing the data. As themes emerged during preliminary analysis, the initial codebook reflecting the evaluation study's key aims, underwent continuous refinement, through discussions between analysts. Codes included in the analysis presented in this paper included *COVID-19, Context, Intervention* and *Implementation, among others*. Analysis involved collaborative interpretation in which research team members engaged in data immersion and familiarization, documenting theoretical and reflective thoughts that developed through immersion in the data, and sharing growing insights about the research topic during regular team discussions. Analytic memo-ing was conducted in parallel, capturing analysts' reflections and interpretations. Through memo-ing, data exploration was enhanced, continuity of conception and contemplation was enabled and communication was facilitated (26). The use of analytic memos created an important extra level of narrative: an interface between the participants' data, the researchers' interpretations and wider theory. Memos also formed part of the summary process, in which analysts articulated interpretations of the data in a more concise format.

The South African Medical Research Council Research Ethics Committee (EC036-9/2020) approved the study protocol. All participants provided informed consent for participation.

## Findings

### Recruitment

In the initial intervention conception, recruitment and demand creation activities were intended to be based at school and Technical Vocational Education and Training (TVET) colleges, at community venues, or through mobile or outreach health services such as HIV-testing events. It was anticipated that implementers would use a variety of strategies to recruit AGYW into the program, including career jamborees, community dialogues, demand creation and community outreach activities conducted at community hotspots. However, restrictions placed on gatherings by the South African Government, as per the Disaster Management Act (Act 57 of 2002) made recruiting new AGYW into the program challenging. As one implementer explained, “*those mass activities… community campaigns… school jamborees, mass events, of course they were not implemented*”.

Outreach and community-based recruitment activities were hampered by lockdown restrictions, social distancing measures, and fear. Additionally, implementers questioned the value and importance of continuing with recruitment activities during this time of crisis: “*We couldn't find more kids on the streets because the instruction was that they should stay at home… We just go to the clinic or we walk down the streets looking for those kids that we can enroll or conduct repeat core (services)... it's like we're selling policies and I don't like it.”* Implementers attempted to continue recruitment through visiting households where AGYW lived, however this was also challenging, and not always acceptable to the community: “*Recruiting becomes very difficult… I go to a family because there are girls, but they refuse to let me in. When it comes to recruiting the girls, COVID has had a huge effect.”*

### Retention

As it was conceptualized, strategies for retaining enrolled AGYW beneficiaries in the intervention included the provision of incentives, reminders through WhatsApp groups, event invitations, linkage to care and follow-ups, home- and face-to-face visits. In order to retain AGYW who had already been recruited into the program during COVID-19 lockdowns, implementers attempted to remain in telephonic contact with beneficiaries listed in their databases. However, due to issues with many AGYW beneficiaries having provided staff with incorrect telephone numbers and inaccessible physical addresses, some of these beneficiaries could not be contacted during lockdown. In addition, the intervention's planned 6-monthly follow-up contact with each enrolled AGYW was negatively affected. One reason provided was that AGYW were unwilling to participate in the follow-up contacts over the phone due to the sensitivity of the content: “*The challenge that we faced was that we then had girls who were then supposed to come back for their 6-month repeat call… we were then told to do these repeat calls telephonically, but the girls would tell you straight out: ‘I am not comfortable talking about my sex life over the phone’.”*

Implementers explained that many AGYW did not re-enroll in the program even after emerging out of Level-5 lockdown, due to a loss of momentum with AGYW losing interest, and the limited programs on offer deterred continued participation. However, there were also instances where migrant households had moved back to their original homestead in another district or province, possibly due to job losses or general economic shocks and stresses: “*When we eventually opened after the lockdown… Most of them showed lack of interest, they no longer wanted to be part of the AGYW program… Most of the girls… moved back to the Eastern Cape and they are no longer in Cape Town, which I understand. But those that are in Cape Town, the majority have been refusing to come back*”.

### Effects of Educational Institution Closures on Implementation

In the design of the intervention, it was anticipated that the core services would be delivered in schools and TVET colleges. Therefore, the closure of educational institutions greatly affected implementation. During the height of first and second epidemic waves, school closures were instituted, in the periods March–June, and again in July–August 2020 (27). During these periods there were no intervention activities offered at schools, dialogues, in-person peer education groups and other group activities. Implementers reported that even after schools began to reopen, they were unable to provide services or extra-curricula activities to in-school AGYW, as schools were prohibited from accepting “visitors” due to COVID-19 regulations. Variations were reported between districts, with some implementers able to return to certain schools from June 2020, whereas in other schools activities were on hold until October 2020, after which most schools reopened. However, in some communities, implementers only returned to schools in 2021, or at the time this study was conducted, had not yet been given permission to re-enter schools to commence their activities: “*When they re-opened in June there was a change… some grades will come this week and other grades will come the following week… There were also some schools which didn't allow us back to their schools because they are saying “there is a circular which is from the district office which says we must not allow any visitors”… when they were back fully in schools, they did allow us to go back to schools… they minimized the interaction between our team and them because of COVID regulations… There were some schools which didn't allow us back.”*

Those implementers who had returned to schools after re-opening described how school staff were hesitant to schedule time for extra-curricula program activities due to the teaching time that had been lost during closures, prioritizing teaching over additional activities. The rotational schedule of students attending school on different days and times also affected implementation. The closure of schools also affected community acceptability, and particularly parental acceptability of the intervention, since the regular means through which implementers engaged with parents through schools were not available: “*Since we usually meet with the young women only without their parents… when we want to meet with them on a certain day and listen to their point of views, we sometimes would call them with their parents in one session. But due to COVID-19 and the fact that we cannot bring people together, it is something that has not happened.”*

Although restrictions meant that regular school-based programs were not able to take place, implementers attempted to modify their program delivery, and provide COVID-19 related support to schools, helping to screening learners, assist with sanitization, and enforce adherence to social distancing measures. This provided implementers with an entry point to the school, and an opportunity to access AGYW for recruitment.

### Effects of COVID-19 on Risk Assessments and Screening

In the initial program design, it was anticipated that in order to design the optimal service plan for each AGYW beneficiary, her specific risks and needs would be identified through a risk assessment process, to be facilitated by an implementer in a private and confidential session using a person-centered counseling approach. However, social distancing measures during lockdowns meant that these risk assessment and screening processes had to be conducted remotely, over the phone. Implementers questioned the appropriateness of telephonic risk assessments, due to the sensitive nature of the questions, particularly those relating to sexual behavior or experiences of abuse. It was also felt that building the necessary rapport and trust with AGYW over the phone was impossible, as was providing necessary support if AGYW were emotionally triggered by the questions: “*Most of our risk assessments are being done over the phone but obviously that has impacted on the quality of our work... you can't see someone's non-verbal communication*”. Implementers also explained that AGYW also could not speak freely and answer openly in front of their parents, when being interviewed telephonically while at home during lockdown: “*Some of our beneficiaries live with parents - they can't be on the phone and talk about the contents of the risk assessment while they are sitting with their parents*”.

In addition to conducting risk assessments and the 6-monthly repeat assessment telephonically during COVID-19 lockdowns, fieldworkers also attempted home visits to access AGYW. However, this brought about various challenges, as it was not always possible for privacy and confidentiality to be maintained in the household. In cases where assessments had to be conducted in the presence of other household members, confidentiality could be breached. Additionally, conflict arose between implementers and parents in situations where parents were not aware that their daughters were participating in the program, as they had not been informed or given their consent. These experiences suggest that the COVID-19 context also contributed to challenges around parental and community acceptability of the intervention, and highlighted insufficient engagement of parents: “*You will meet with parents with problems, asking you how you allowed her kid to enroll without his or her consent?... that they don't want their children enrolled in the program… If you're going to ask her sexuality questions and about boyfriends, they can't be free because of the presence of the parents. And if you ask to speak in private with the child, the parent refuses and asks “what is it that you are going to discuss with the child in secret, that I cannot hear?”*.

### Effects of COVID-19 on Biomedical Services Provision

Included in the intended intervention design were comprehensive biomedical services that were to be delivered at mobile or fixed clinics in or near schools, and in communities. Contraceptives and pre-exposure prophylaxis (PrEP) were to be provided to AGYW either directly by SRs or through government clinics. Respondents explained that during the COVID-19 pandemic and lockdowns, AGYW access to biomedical services was disrupted, particularly contraceptives, HIV testing and PrEP, raising concerns that teenage pregnancy, HIV and STI infections, would increase: “*COVID disturbed everything… when the COVID started we were not allowed to do the community testing… we were only allowed to go to the clinics… so we were not able to reach the girls in the community… The girls couldn't have a chance now to come to us because of the limitations… and as a result we got a lot of girls that were defaulting now on their appointment days for the family planning”*.

At the start of the COVID-19 pandemic services were halted, while implementers were developing a strategy to adjust programs to the changing context and to apply for permits to keep offering services. Later on, some implementers resumed offering biomedical services in mobile clinics, however, accessing AGYW proved to be challenging. Events involving HIV-testing had to be canceled due to COVID-19 restrictions on gatherings: “*We usually do events for the girls, for mass (HIV) testing and some sort of dialogue... They had to postpone that until further notice, until the lockdown was lifted”*. While events were prohibited, implementers attempted to provide HIV testing at their mobile truck, however some AGYW believed the mobile truck was for COVID-19 testing and were afraid to approach it: “*During the first lockdown, we all stopped (providing services)… Then a go ahead was given later on, and we continued with the program… It did have an impact because the turn out was not as usual… some were even afraid of the truck, thinking it is there for COVID test. They do not even want to pass near it”*. Providing program services in the clinic setting was especially challenging during the height of COVID-19, as AGYW were fearful of going into public health care spaces, due to fear of infection: “*If they (AGYW) have to test for HIV they are scared to go there (to the clinic) … Because remember if you go to these health facilities you might get COVID-19… the reason at times they don't want to access the services is because they would say they are scared to be infected”*. In addition to fear of infection, AGYW were also hesitant to go to clinics as they expected increased waiting times due to COVID-19 protocols: “*After recruiting girls, you refer them to the clinic. (But) they won't go because the clinic does not operate normally like before COVID-19”*. Implementers also explained that AGYW were often unable to get to clinics due to public transport restrictions.

There were also cases reported where staff had contracted COVID-19 or had contact with COVID-19 patients and therefore needed to self-quarantine, which disrupted biomedical service delivery to AGYW: “*During COVID… we were staying home, after having some contacts (with COVID patients), some of the children missed their days because we were not there. Some went to the clinics and they chased them away. Some didn't even attempt to go to the clinics when it was their day, especially for family planning”*.

### Effects of COVID-19 on Behavioral and Structural Services Provision

As part of the layered behavioral and structural services provided included in the intervention, programs included various group-based activities, for example psychosocial counseling and support groups, peer education sessions, self-defense classes, and recreational activities. However, as a result of COVID-19 restrictions on gatherings and social distancing regulations, group sessions could not be conducted: “*Our trainings were affected because there were limitations in terms of the number of people required in any gatherings… most of our trainings were put on hold”*. During the height of first and second waves, only core intervention services were being provided, and therefore none of the community dialogues, peer education groups or other group activities were able to take place, which caused disappointment amongst AGYW beneficiaries: “*We did have a plan of activities… to do public speaking and debates with girls from other towns. But we couldn't do it because of COVID-19, and the kids has been so excited”*.

Even after lockdown regulations were eased and group sessions were allowed to proceed, the limited numbers of AGYW allowed per group activity deterred AGYW from participating: “*We would say we have drama clubs and drama groups, but it is limited to 15 (participants)… and then the girls lose interest”*. Despite the easing of restrictions enabling group sessions to recommence in reduced numbers, social distancing requirements and regulations meant that certain aspects of programs such as self-defense classes which involve physical contact, could not be delivered at all: “*Our groups, when we started, when we were on Level 2 or Level 1, they were about 10 (people)… for the self-defense classes, we had 20 and more, and now they had to be cut down to 10 per group. But they could not even practice some skills in self-defense classes… they could not demonstrate (physically) what they needed to do”*.

Respondents also described the challenges they had experienced when attempting to re-start group sessions after restrictions were eased, as some AGYW were resistant to follow COVID-19 safety procedures or wear masks, due to misconceptions or a lack of information: “*The majority of these girls don't want to wear a mask… you have to provide them with a mask and then they don't know or understand the significance of wearing a mask. Or sanitizing… when you sanitize them, they will laugh at you… They think that COVID it was for those who were staying in (another province), not here”*. COVID-19 denialism was also evident in some communities, which meant that AGYW were resistant to adhering to safety protocols: “*They don't understand COVID-19 at all… when we were still in Level 5, they were not wearing masks… they don't take COVID-19 seriously, they think it doesn't exist”*.

### Effects of COVID-19 on Referrals to Government Services and Other Service Providers

After completing the risk assessment and screening process, implementers held consultations with each AGYW to develop an individualized service plan and discuss the services available to her through referral to various community based and governmental service providers. The approach of the intervention was to refer AGYW to leverage existing services in the community rather than set up duplicate, and less sustainable services.

Referrals to government departments to deliver various services to AGYW were negatively affected by the COVID-19 pandemic. As already described, AGYW were hesitant to go to clinics, due to fear of infection and increased waiting times due to COVID-19 protocols and health workers' task-shifting. Respondents also explained that referrals to the Department of Home Affairs and the DSD were negatively impacted, and service delivery efficiency declined: “*COVID also became a blanket term to paper over very systemic inefficiencies everywhere, with schools, with clinics, with police stations, with funders, with every person, every structure that you engage with. COVID became… a convenience”*. Failures to refer to Home Affairs impacted AGYW livelihoods since access to social grants was affected. The intervention's “back-to-school” component was affected by difficulties in accessing the necessary documents such as identity documentation for AGYW to be accepted back into schools once they reopened. Implementers also described several barriers to effective collaboration with DSD due to COVID-19: “*DSD... they said that they'll provide support but it hasn't materialized… COVID has impacted how we can engage more fully and the offices are not as open to the public as it maybe could have been in the past… I can't say that we have a relationship that's working as yet”*.

Overall, referral processes with government departments were complex, with context specific issues and idiosyncrasies in each sub-district, dependent on the nature of established working relationships with key personnel in government departments, to whom referrals could be directed to. The success of referrals was dependent on these relationships. In cases where the key contact person passed away, or left their job, it would create new challenges and delays in the referral processes, as implementation teams had to re-establish the referral pathways with new people. Moreover, virtual operations and limited operation periods of government departments also presented further referral challenges.

### Program Adaptations to Respond to the COVID-19 Context

Responsive modifications and adaptations to the recruitment strategy and intervention design to respond to the challenges of the COVID-19 context included moving away from holding events and group sessions: “*We were supposed to have groups, career jamborees… we had to and shift… try and redo them in a different manner”*. Career jamboree events were replaced with the provision of printed information, education and communication and recruitment materials, and smaller school-based events: “*With the career jamboree, we have to change from the original jamboree. We have developed a jamboree booklet instead… if we can't (be in schools) … we print user friendly booklets so that they can have information regardless of COVID-19 happening”*. Another successful strategy for engaging with AGYW created in response to COVID-19 involved recruiting in mobile units/vehicles inside communities and making use of speakers and a loud hailers to minimize personal contact: “*We took the company car… we went into the community... we were calling them on a loud hailer… playing music… that's how we attracted them (AGYW)… if you are sitting there, just waiting for them to come, it's not going to work”*.

### Offering Remote/Virtual Support

Since group sessions were no longer possible, as many services as possible were offered remotely: “*For COVID we had to now move from physical sessions to telephonic interventions…”*. As an adaptation to the context of lockdowns and social distancing, implementers made efforts to remain in contact with AGYW beneficiaries over the phone, offering psychosocial support, providing SRH information and engaging them on social media platforms such as WhatsApp groups: “*We had to stop going out to the community when the lockdown started in level 5… so we were calling the girls to check up on them and find out if they're okay… and sending them information like for sexual reproductive health… sending things via WhatsApp”*. During Level 5 lockdown, offering one-on-one telephonic support and support via WhatsApp groups and Zoom (in limited cases) was one way of ensuring some continuation in contact with AGYW, offering psycho-social support: “*It made our job simple when they (management) started providing us with airtime to call the children and set appointments with them, especially if the case is sensitive and needs to be attended at quick then you call them”*.

One challenge in offering services telephonically was that many AGYW did not have their own cell phones, but shared one with other family members. However, implementers found ways of accessing AGYW despite these challenges: “*It's still a challenge but we are making it work… What I usually do, I call and tell the parents that I'm the social worker based in schools… I'm calling just to check up on them (AGYW)… that's how I get them (AGYW) on the phone”*. The creation of WhatsApp groups was also used an engagement strategy: “*The best thing was that we opened a WhatsApp group engaging with these learners and getting them in the community… even those who didn't have cell phones, they were using their parents' phones”*.

### Offering Door-to-Door Services

During Stage 5 lockdown, some implementers managed to acquire special permits to operate during lockdown, and could continue doing home-visits to AGYW: “*We were given the permits, but it was hard you know, working with the permit and going door-to-door to people's houses. Some people were really worried about COVID but we were also going around with COVID tools (PPE equipment)”*. To circumvent challenges brought by the closure of schools and tertiary institutions, and the lack of AGYW access to clinics during lockdowns, to mitigate discontinuation of PrEP, contraceptives and SRH service provision, some implementers were providing SRH services and counseling services door-to-door: “*AGYW were struggling to come and receive these services, so we were doing door-to-door… Especially for the ones that are on PrEP, so they received follow-up medication… we were doing door-to-door testing… and then we did one-on-one counseling”*.

### Successes and Challenges of Responsive Adaptations

Despite the innovative responses of implementers and attempts to adapt services to be provided remotely or door-to-door, not all adaptations were easy to implement or successful. For example, implementers described various challenges in providing psycho-social support over the phone. In some instances, reaching AGYW at home also created conflict with parents and other household members, who were not aware that the AGYW were enrolled in the program. AGYW also experienced discomfort discussing sensitive issues over the phone, or those suffering from abuse in their homes, may not be safe to disclose over the phone: “*They can't give psychosocial support on the phone. It's easier when you see somebody because then you see the body language and the expression. They are at home, they are uncomfortable speaking… I mean can you imagine if the uncle of the dad is abusing this girl at home and now she has to talk on the phone in front of everybody about the issue”*. Ethical issues of not being able to provide sufficient support over the phone were raise: “*In terms of counseling… it's difficult to have a session with a child on the phone because you can't give that support to them... They're not comfortable to talk in front of their families especially the kid which is being abused at home. We can't talk to the child… she doesn't want parents to know, so it has affected us in a very negative way because now we are unable to provide AGYW with the support that they need”*.

Some AGYW beneficiaries did not respond well to this new medium of communication and were either unable to participate or unwilling to participate in online or WhatsApp groups or to receive psychosocial support telephonically. In addition, some AGYW beneficiaries did not have either a personal phone or more commonly, access to sufficient data: “*Some (AGYW) don't have cell phones, the ones that have phones don't have airtime to make a call and ask for help or support with a certain issue”*. Even when implementers started using data-free sites, some AGYW had challenges accessing them: “*They (AGYW) don't always have data access, so even WhatsApp, some of them couldn't read it (the information we sent)... because they couldn't get to the free sites for network access”*. Implementers felt that they were demanding too much of AGYW beneficiaries to spend precious data on program activities: “*Some of them (AGYW) had a challenge with data… they say that we don't have enough data to be always chatting to you on a daily basis”*. Limited data and network access meant that retaining AGYW in the WhatsApp groups was challenging: “*WhatsApp does not work. Facebook very few of them (AGYW) are on Facebook and some of them say: ‘Hey, you want to reach me on WhatsApp but you don't buy me data and I don't have Wi-Fi at home’… You form WhatsApp groups with 20 people in it and before you know it you only left with five”*.

Due to AGYW expressing a preference for in-person face-to-face services, some implementers arranged to continue to provide face-to-face psychosocial support when this was possible, even though group sessions were halted. Some one-on-one psychosocial support services, where AGYW could see a social worker, were arranged through scheduled appointments: “*The feedback from the AGYW has been that they don't want to talk over the phone. They actually want the contact face-to-face contact. So, the minute we were able to set up the counseling, I think it was level three, we did face-to-face psychosocial support”*.

As many of the intervention's services were not available during lockdown, some implementers felt that telephonically contacting AGYW prioritized reaching recruitment targets over service provision, and therefore negatively impacted their ability to offer quality care: “*To be honest, even though we did our best, I do not think that we did justice, we did not provide a high-quality service to our beneficiaries. They also did not access the services; none of the services were available during that time. Especially on level 5, there were no services available; so, it was just chasing numbers to be honest… We contacted the young girls and we said we would refer you, but we never did”*.

Offering door-to-door services also proved challenging for various reasons. In many cases, AGYW and their family members were wary of inviting fieldworkers into their homes due to fear of being infected by field staff, which limited the effectiveness of door-to-door visits. In some communities, rumors circulated that program field staff were actively spreading COVID-19 in the community by doing home visits: “*You go to a place and knock (on the door) looking for the child, they think that you are bringing COVID to their homes. They did not trust us at all, to such an extent that it was rumored in some places that we are the ones that bring it (COVID-19) to their homes”*. There was a mutual fear of infection between and amongst families and implementers: “*We were doing door-to-door, but they were chasing us. We tried to make some rapport and make some friendships so that they would trust us… it was so tense! Everyone was scared of everybody, even the co-workers… The lack of trust between the community and us… they think we are the carriers of the COVID to them”*. Staff themselves were also reluctant to conduct outreach activities. Fieldworkers were concerned about contracting COVID-19 during outreach activities from co-workers or from AGYW and the community: “*We couldn't do home visits because we were afraid of getting COVID-19 and the people we were supposed to visit, were also afraid that we will infect them with COVID-19… We were afraid of one another”*.

Amongst all the challenges brought by the COVID-19 context and restrictions, there were also examples of positive outcomes. For example, one positive adaptation described by implementers was the way in which the new working protocols forced an improvement in staff use of and familiarity with online systems for data management, reporting and virtual meeting platforms. These changes were reported to have lasted beyond lockdown, helping to improve work processes: “*The positive is that we innovated as a team, we moved all our work online. People had to quickly adjust to how to operate on Google Drive and how to edit work online… now everything we learnt during COVID is stuck into our work process and has helped us to better manage our data… right now I can tell you what is happening in our data because of those… processes and innovations… for me it is exciting because that is where the world is moving… Google meetings and Zoom meetings”*.

### Responding to Community Needs During COVID-19

Some of the implementer respondents expressed views that in general, program funding was not flexible enough to allow responsive real-time adaptations and modifications to the programs. According to some respondents, funding arrangements and budgets had been developed before the pandemic, and in some cases it was not possible for finances to be reallocated to resources and services that would meet specific needs in the pandemic environment, for example providing data to AGYW to access remote services, investments to offer programs virtually, or provide relief packages for AGYW households. Despite the restrictive financial set-up, in some cases implementers tried to be responsive to the increased economic stresses experienced by households. As implementation continued during the pandemic, dedicated COVID-19 response budgets and funding were provided to implementing organizations, to allocate to relief efforts: “*We tried to get food parcels, food vouchers, sent to their phones where possible, via other networks… because we're a community development organization, that's what we usually do, so we could also do other things to supplement the program, which was helpful”*. Some implementers were directly arranging for food parcels and vouchers to be delivered to AGYW households during lockdown: “*We were giving them food parcels during COVID… see who has those challenges, that need serious attention and refer them to a social worker, and they will get a food parcel”*. Others were making referrals to social workers and DSD so that AGYW in the poorest households could receive food parcels and psychosocial support: “*We engaged with the Department of Social Development, where they can assist… Even though we didn't have the capacity to give them the food parcel and what not, but at least we had other stakeholders that are working hand in hand with us, so that they can assist those girls (in need)”*.

Implementers also assisted clinics and schools to screen for COVID-19: “*During the pandemic, we have partnered with the Department of Health, so that we can be in the community and help with screening. During screening we enroll the kids into our programs”*. Implementers also worked with the Community Action Networks (CANs) that were formed during COVID-19 to support local communities. Fieldworkers would use these opportunities to recruit AGYW into the program: “*Some of our team has been working with the Community Action Networks that were formed during COVID and so they have been sourcing the young women, they provide the food and we do the recruitment and engagement and sharing ideas”*. Field staff were providing information and education to AGYW and their families, equipping with them the knowledge needed to protect themselves against COVID-19 infection: “*We were teaching them how to wear a mask, how to wash their hands and we told them about all of the regulations that we should follow for COVID-19”*.

### Logistics, Staff Management, and Morale During COVID-19

Respondents described various ways in which they had tried to manage logistics and support staff to keep implementing during COVID-19 in face of the challenges. Implementers described a multitude of barriers to reaching program targets due to COVID-19, which in turn negatively affected staff morale; staff felt that they were not valued, despite facing difficult and often dangerous working conditions, crime and risk of COVID-19 infection: “*The staff had to work twice as hard to meet targets… you cannot meet in big groups anymore… People don't want them in their homes… nobody's safe”*. Levels of stress amongst implementers increased as work challenges escalated: “*COVID-19 destroyed our lives!... We went to work after that very strict lockdown… We were struggling to reach the numbers because the children are not in the streets, we were not allowed to go to school because the schools are closed… (staff) were stressed because they couldn't find the children… a lot of staff contracted COVID-19... (the community) told others that we will bring COVID-19 to them”*.

Transport logistics for field teams were complicated and prohibitive during lockdown, since regulations limited the number of people who could travel together in a vehicle: “*Previously, we were sharing the car but then our manager said, ‘only 3 people in a car’. So, it was not easy, we had to travel with 3 people to a site and then come back with the car, to fetch another 3 people, because we are more than 3 in our team… it was just consuming time and consuming petrol… it started to be easier when we were allowed 5 in the car”*.

Managers explained that it was difficult to keep staff motivated during lockdown, since the working environment had become challenging and, in some contexts, unsafe. Staff were worried about catching COVID-19 during outreach activities and in-person services: “*We were all worried about getting COVID, but we all need food on the table, so we went out to do what we have to do”*. Staff felt under pressure to meet the targets that seemed unreasonable given the pandemic context: “*It was quite hard to keep staff motivated to do phones and WhatsApp calls to the young women because a lot of the (phone) numbers are wrong, we couldn't find the girls and that did affect staff morale quite a bit. But then coming back we had to push really hard to then catch up on target, because the quarter over COVID… our total reach was maybe 29 or 30%”*.

There were also instances in which staff themselves fell ill, their families were affected or they had been in contact with COVID-19 while some staff members had to be isolated/quarantined. Therefore, implementation was challenging, given that teams were not always complete: “*It affects our stats (numbers) because we have to stay home for a while when we have contacts (with someone who is COVID-positive)… This week someone is ill and then the next week someone is ill, so we were not a complete team”*.

In any multi-site, complex intervention, staff training needs are complex and on-going. The COVID-19 context added further complexity, with teams having to adapt to the changing context. Respondents described the challenges adapting trainings to an on-line format. Many staff did not have access to computers and internet at home, and it is difficult to ensure the efficacy and quality of training when delivered remotely. Some of the more technical aspects of clinical training need to be practical and in-person.

### Implementer Recommendations for Implementation During COVID

In light of perceptions that program funding was not flexible enough to allow implementers to adapt the program to the COVID-19 context, respondents felt that they would benefit from having more flexible funding in order to best respond to the changing context and bring some program components online: “*The budget also doesn't allow for some of the things that we would like to do; so, a bit of flexibility with the budget so that we can move some of these things online… this program is very rigid! There is no room for flexibility… there is a saying that goes: ‘adapt or die’. But in this case, it is the program that is not adapting and it is the girls that are dying”*. Implementers described the ways in which online platforms could better be utilized to deliver and provide psycho-social support that do not involve costs or data expenditure for beneficiaries: “*We need to get a way of communicating with them (girls) maybe via Skype but in such a way that they don't get charged on their phones”*.

In contexts where AGYW homes are not safe or confidentiality cannot be assured, it was felt that services are best provided outside of the home if COVID-19 restrictions allow and if safety protocols can be followed: “*For the services to continue, you should remember that for some kids, the problems are right at home, the abuse is at home where she lives… if we can find a way of continuing with the services despite COVID, that would be good… when COVID subsides there will be more problems, even the child that was better will be in more deep pain”*.

Implementers described the need to be sufficiently prepared and aware of how programs can be adapted and what measures can be put in place at different levels of COVID-19 lockdown: “*We have to look at what elements of the program can be done online and what is the best way to go about it… we also have to look at the different levels of lockdown and how we are going to respond… if you are at level 1, this is how you need to implement, and if you are at level 3, this is how you need to further escalate, and then if you go into full lockdown, which is level 5, this is what you are able to do, and this is how you should do it”*.

## Discussion

Using the concept of health system resilience to frame our findings, we describe how a South African combination SRH intervention for AGYW was impacted by the COVID-19 pandemic and related restrictions, provide details on the various responsive adaptations that were made to implementation, and make recommendations (summarized in Table 1). As evident in the narratives of implementer respondents, the COVID-19 restrictions and lockdowns, including the closure of schools, and social distancing measures, have had significant impacts on the implementation of the intervention for AGYW, undermining service provision, impeding recruitment and retention of AGYW beneficiaries, and heightening issues around community acceptability of the intervention. Mid-stream adaptations to respond to the pandemic context included offering remote support through telephone or digital platforms, providing services through door-to-door home visits, the provision of food parcels or referrals to AGYW in need, and collaboration with community based organizations. Adaptations made in response to the challenges implementers faced in continuing to provide services to AGYW beneficiaries during COVID-19 and the lockdowns, varied in their success, providing valuable lessons learned for future preparedness for crisis scenarios.

**Table 1:** Adaptations table.

**Area of adaptation**	**Reasons for adaptation**	**What could no longer occur**	**Adaptation/modification made**	**Successes and challenges of adaptation**	**Recommendations**
Recruitment and demand creation	Restrictions on gatherings, social distancing measures	No community-based recruitment events	- Household door to door visits - Events replaced by printed materials	Challenges: - Fear of infection and safety concerns amongst both families and staff during household visits	- More effective use of online platforms to deliver and provide psycho-social support that do not involve costs or data expenditure for beneficiaries. - Provide psychosocial services outside of the home if COVID-19 restrictions allow and if safety protocols can be followed. - Provide AGYW with airtime and data. Provide toll-free phone helplines, zero-rated instant messaging and data-free websites to provide SRH information, health and programme activities to those who need it. - Provide skills training to enable AGYW to engage and communicate safely and effectively in digital spaces. - Community engagement, supportive relationships with civil society and community based organizations. - Collaboration, proactive relationship building and meaningful engagement between the private sector, civil society and government service providers, through formalized multisectoral partnerships - Conduct individual, social, and environmental risk factor assessments for individual AGYW before conducting SRH, psychosocial or risk assessments telephonically, with special precautions taken to ensure privacy, safety and confidentiality when working with young people deemed “at risk”.
Programs offered in educational institutions (schools and TVET colleges)	Closure of educational institutions	No non-curricular activities permitted in classroom contact time When schools were open, classes reduced to 50% capacity	- Helping with COVID-19 screening of learners - Assisting with sanitization awareness - Helping to enforce adherence to social distancing measures	Challenges: - Resistance from school staff to extra-curricular programme activities due to lost teaching time - Limited opportunities to engage with parents Success: - Provided entry point to schools, and opportunity to access AGYW		
Risk assessments/screenings, follow-up assessments	Social distancing measures, lockdowns	No face-to-face screening or limited face to face interactions	- Screenings conducted remotely - Attempts to follow up with beneficiaries telephonically	Challenges: - Fear of infection during household visits - Inadvertent disclosure of AGYW participation to parents through shared household phones and lack of privacy in homes, creating conflict in homes - Problems with telephonic contact due to database issues - Sensitive nature of questions, not possible to build rapport or provide necessary support over the phone		
Health service provision	Restrictions on gatherings, social distancing measures, lockdowns, restrictions on public transport	Limited access to clinic-based service provision of contraceptives, HIV testing, PrEP. No mass HIV testing events	- Mobile services offered out of mobile unit - Outreach services door to door	Challenges: - AGYW fear clinics due to infection and waiting times - AGYW fear of mobile units - Difficulty getting to facilities due to public transport restrictions, staff shortages due to infection and having to quarantine Success: - Continuity of some health service provision to AGYW willing to access approachable mobile units and outreach services		
Behavioral and structural services	Restrictions on gatherings, social distancing measures, lockdowns	No group-based activities, support groups, peer education sessions, self-defense classes, or recreational activities	- Offering psychosocial support and counseling remotely or door to door - Providing SRH information to and engaging with AGYW on social media platforms, WhatsApp groups	Challenges: - AGYW resistant to follow COVID-19 safety procedures or wear masks - AGYW loss of interest in programs - AGYW without personal phones or sufficient data - Limited network in some areas		
			- Some organizations offering data bundles, toll-free counseling hotlines, and reverse billing support service - One-on-one counseling through scheduled appointments - When permitted, small group activities	- AGYW discomfort discussing sensitive issues over phone - Not possible to ensure confidentiality over phone Success: - Improved staff literacy with online platforms and remote working methods - Continuity of programs for AGYW by conducting programs and services remotely	

Implementers attempted to be responsive to the challenges that AGYW beneficiaries and their households were facing during the COVID-19 lockdowns, with increased levels of food insecurity and hunger, compounded by loss of income for families, through the provision of food parcels or referrals to the DSD. However, despite the later addition of the COVID-19 response budget, respondents expressed frustration that the program funding was inflexible, and did not allow for quick and responsive program adaptions to the pandemic context. Respondents felt that more flexible funding could have allowed responsive reallocation of resources to meet contextual needs, for example providing data to AGYW, investments to offer programs virtually, and material support such as food relief packages for AGYW households. Flexibility in financing mechanisms, allowing for reallocation in response to shifting priorities has shown to be a key ingredient for ensuring health system resilience in times of crisis (28). The COVID-19 pandemic situation has demonstrated the critical importance of “learning while doing”, and models that allow interventions to make real-time adaptations and adjustments to respond to emerging evidence and urgent challenges brought on by or exacerbated by the pandemic, including access to psychosocial support and SRH services, and food insecurity (25, 29). Future interventions should improve the reporting of and capturing of details regarding the adaptations and modifications made using methods such as FRAME, to facilitate learning in the process of modifying interventions, and ensuring effectiveness of implementation, scale-up, and sustainability (22). This would enable researchers and implementers to compare and learn from adaptions, and their successes and challenges, across interventions and settings.

One strategy that implementers employed in an attempt to ensure continuity in service provision was through outreach services. Some of the biomedical service providers had acquired permits to operate during lockdown, and attempted a door-to-door continuation in service provision to AGYW. However, there were several challenges with this approach, particularly during the height of the first and second waves of COVID-19. AGYW and their families were wary of inviting fieldworkers into their homes during this time. Staff were also reluctant to conduct outreach activities due to both potential infection and safety concerns. In addition, conducting home visits carried the risk of causing AGYW discomfort through inadvertent disclosure of program participation to family members. The lack of engagement with parents during the early stages of the intervention was demonstrated by the fact that many parents were only made aware of their daughters' participation in the program when fieldworkers visited their homes to conduct outreach and follow-up activities. Implementers also highlighted challenges in accessing AGYW in homes where abuse and violence occurred. Future programs should take into consideration the way in which any intervention that reaches into users' homes and personal spaces could also entail particular risks for AGYW (30).

One key strategy for adapting services to the COVID-19 pandemic and lockdown context and ensuring some level of service continuity was through offering as many of the services as possible remotely. The use of telephonic and online platforms to provide support and information during the pandemic was viewed as a pragmatic and feasible way of ensuring continuity of support, echoing findings from a survey conducted by the World Health Organization showing that the employment of telemedicine as a substitute for in-person consultation was one of the most common approaches globally to mitigating the impact of COVID-19 on routine services (17). However, respondents in our study felt that some program elements were inappropriate for remote delivery, for example conducting risk assessments or providing telephonic counseling, were described as problematic and inappropriate due to the sensitive nature of discussions. Respondents detailed the various risks of AGYW disclosing risk behaviors over the phone, particularly when using shared family cell phones in households where abuse occurs. Implementers explained that AGYW were also reluctant to discuss their participation in the program or their sexual behaviors over the phone, due to fear that parents might overhear the discussion. SRH is a sensitive area, requiring high levels of confidentiality in order to maintain privacy and safety of AGYW; breaches in confidentiality or inadvertent disclosure can result in conflict or loss of support from parents/caregivers, or even violence (30). This is particularly the case for issues such as AGYW use of contraceptives or PrEP, which parents may not condone (30). There is strong evidence that risk assessment and psychosocial support interventions can be conducted safely and effectively through telehealth means, and that in some cases young people even disclose more information over the phone than they typically would during in-person sessions. However, the bulk of this evidence comes from high-income countries where digital literacy and access to technology are likely to differ from the South African context (31). In resource restricted settings where phones are likely to be shared between family members, the potential for SRH interventions to deliver information on contentious topics or relating to sensitive personal information telephonically needs to be carefully considered. There may be potential to inadvertently exacerbate risk in cases where AGYW have restricted access to safe and confidential spaces, and live in home environments where they cannot safely seek support and advice telephonically (30, 32). Individual, social, and environmental risk factors for individual AGYW need to be taken into account when conducting SRH, psychosocial or risk assessments telephonically, with special precautions taken to ensure privacy, safety and confidentiality when working with young people deemed “at risk” (31, 33).

Given the context of restrictions imposed by COVID-19 regulations, the need for remote support has increased. The use of online platforms to provide support and information during the COVID-19 pandemic was a critical means of ensuring continuity of support. The potential for online support or “e-mentoring” in resource-constrained settings has been highlighted elsewhere (34). Formal e-mentoring via digital platforms has been shown to be a viable and pragmatic solution for ensuring continuity and access to psychosocial support for adolescents and young people in the COVID-19 pandemic context (34). Digitally based services can work toward tailoring service provision toward the choices, preferences, and agency of AGYW as users (13). Digital heath, support and mentoring platforms could also have the potential to support AGYW to build skills for self-care and increase autonomy in health-seeking behavior. AGYW can be empowered with the tools, information and opportunities to identify their own health needs, and access appropriate interventions enabling them to take an active part in decision-making about their own health, thereby promoting self-reliance and autonomy (14, 33, 35, 36).

Evidence showing the potential for digital health and support to advance accessible, equitable, affordable, quality health care and psychosocial support to populations at scale is strong (14, 33). However, as illustrated in the findings from our study, where respondents described a key barrier to AGYW accessing remote support being the requirement of paying for data/airtime, AGYW in these communities may face disproportionate difficulty in accessing mobile and digital technologies (33). Our findings have shown that in addition to offering programs online, AGYW need to be provided with airtime and data to ensure these costs do not need to be absorbed by struggling households, especially with the high data costs in South Africa. Providing toll-free phone helplines, zero-rated instant messaging and data-free websites would enable free and easy access to important accurate information about SRH, education, health and program activities to those who need it. Notably, in the period just after data was collected for this study, some of the implementers had started providing toll-free counseling hotlines, and deployed a reverse billing support service offering information on topics including COVID-19 and the community response, adherence to medication (for HIV, TB, STIs and non-communicable diseases), gender based violence, accessing post-violence care services, and promoting safer sex and harm reduction practices. Assisting young people in resource-constrained settings with technology to access digital support, designed in a user-centered manner that considers the digital literacy skills and technology accessibility of individuals, is also a prerequisite (33). In order to promote universal access to digital technologies and address inequities based on gender and socioeconomic factors, there needs to be increased investments in digital technology and internet infrastructure, alongside programs to improve digital literacy amongst the most marginalized and vulnerable AGYW (13, 14).

The COVID-19 pandemic has demonstrated the importance of flexible approaches to health care delivery and accelerating the introduction of innovative service modalities. We need to ensure that the urgency of responding to the pandemic crisis does not impede change processes in the non-pandemic setting (28, 37). The rapid shift toward providing digital health interventions has provided an opportunity to address some of the existing geographic and socio-behavioral barriers in accessing SRH and psychosocial services, and innovatively transform existing health systems and improve services and healthcare (7, 38). The benefits of remotely delivered technology-driven services may be of particular benefit to the most vulnerable, marginalized, and hard-to-reach adolescents and young people, who currently face many barriers to accessing care and support (7, 38). For AGYW, this shift may enable increased access to appropriate and tailored SRH care, including HIV prevention and treatment, and contraceptives (7).The COVID-19 context has increased momentum for innovative health system adaptations, and illustrated the potential for digital technologies to address some issues of equity and access to SRH services. In the process, AGYW have been supported to access healthcare conveniently and safely, therefore contributing to positive SRH outcomes. However, not everyone has equal access to the digital space, or has the necessary skills or digital literacy to effectively engage with and use it (7, 13, 30). In addition to the provision of technology, with which to access these online platforms, it would be beneficial to provide skills training to enable young people to engage and communicate safely and effectively in these digital spaces. This would ensure that digital infrastructure can be expanded in a way that ensures that digital inequalities are not exacerbated (34, 38).

Our findings illustrate various challenges relating to ensuring parental and community acceptability and buy-in of the intervention linked to the context of COVID-19 lockdowns, as opportunities for community events were limited. However, one successful mechanism for engaging with communities during this time was through implementers' collaboration with Community Action Networks (CANs). CANs are self-organized neighborhood networks comprising local residents, businesses and civil society organizations, engaged in rapid, community-led responses to COVID-19 (39). Community engagement can help to bolster resilience of health systems and services, and community health workers can enhance a system's capacity to respond to crises situations such as COVID-19 (40). However, as seen in the narratives of respondents in this study, the health and safety of community based workers during pandemic situations is a critical consideration. Supportive relationships with civil society and community based organizations, such as intervention implementers' collaboration with CANs, can help to ensure continuity in service provision, and rapid response to community needs (39, 40). Collaboration between key stakeholders including coordination of activities, proactive relationship building and meaningful engagement between private sector, civil society and government service providers, through formalized multisectoral partnerships may help to limit disruptions in health service access, and build health system resilience in the longer term. This approach can assist in safeguarding continued access to services even during crises such as during the constraints of the COVID-19 pandemic (14, 23, 28, 32, 37).

A limitation of this study is that data was collected during the early phase of the grant period. As there was a staged roll-out of various services and interventions, not all the intervention components were yet being widely implemented at the time of the data collection. Since data collection was conducted at one time-point in the implementation of the intervention, as per the design of process evaluation studies, we were not necessarily able to capture all the mid-stream adaptations made by implementers. To address this limitation in the evaluation, implementers were provided with the opportunity to provide feedback on the evaluation, and to furnish details on any mid-stream adaptations or modifications that had not been captured during the evaluation process. Finally, details on intervention adaptations were collected after the fact, and not systematically recorded during implementation.

## Conclusions

Evidence of the disruptions to AGYW healthcare access during the COVID-19 pandemic and lockdowns, and the national health system crisis brought about by the pandemic, highlight how critical it is for interventions, programs and health systems to be flexible enough to respond and adapt in order to remain resilient in the face of multiple stressors, and for service providers to adopt sustainable and innovative strategies and platforms to ensure the continued delivery of SRH and psychosocial services to AGYW in South Africa (14, 19, 41). It is important to develop innovative ways to ensure AGYW's access to health, social protection and educational interventions during situations like the pandemic. Without continuity of SRH and psychosocial support services during emergencies such as the COVID-19 pandemic, any gains achieved in SRH indicators will be reversed, leading to rising HIV infection, teenage pregnancies and other negative health outcomes, and thus further exacerbating the vulnerability of AGYW (19). The AGYW intervention provides a model for assessing health system resilience, and the ability of actors within this system to adapt and respond to a crisis situation. Evidence shows that resilient health systems are those that entail comprehensive responses, and include elements to address health and wellbeing, but also social and economic challenges, at the individual and household level (25). From the initial conceptualization, the intervention was designed to be comprehensive, comprising biomedical, behavioral and structural services. By modifying aspects of service provision, implementers were able, to a certain extent, to provide context appropriate services and support to AGYW beneficiaries. The findings from our study can help to inform efforts to provide uninterrupted and continuous services to AGYW, ensuring that SRH interventions and psychosocial programs can withstand pandemic situations.

## Data Availability Statement

The datasets presented in this study can be found in online repositories. The names of the repository/repositories and accession number(s) can be found below: https://www.samrc.ac.za/intramural-research-units/HealthSystems-HERStory.

## Ethics Statement

The study protocol and methods were reviewed and approved by the South African Medical Research Council Research Ethics Committee. The participants provided informed consent to participate in this study.

## Author Contributions

ZD was the principal study investigator of the qualitative study component, performed analysis of data, and led the manuscript writing. BB was a co-analyst and contributed to writing the manuscript. CF was a co-analyst and conducted reviews of the manuscript. KJ and DG were co-investigators of the study and conducted reviews of the manuscript. CW, KM, and AA were involved in implementation of the intervention, and provided valuable input on the manuscript. CM was a co-principal investigator analyst and contributed to writing the manuscript. All authors contributed to the article and approved the submitted version.

## Funding

This research has been supported by the Networking HIV and AIDS Community of Southern Africa (NACOSA).

## Conflict of Interest

The authors declare that the research was conducted in the absence of any commercial or financial relationships that could be construed as a potential conflict of interest.

## Publisher's Note

All claims expressed in this article are solely those of the authors and do not necessarily represent those of their affiliated organizations, or those of the publisher, the editors and the reviewers. Any product that may be evaluated in this article, or claim that may be made by its manufacturer, is not guaranteed or endorsed by the publisher.

## Footnotes

^1^South African Government Gazette 43599, 7 August 2020.

^2^More details on HERStory2 study can be found at https://www.samrc.ac.za/intramural-research-units/healthsystems-herstory
